# Simultaneous reconstruction of the bone and vessels for complex femoral defect

**DOI:** 10.1186/s12957-016-1037-8

**Published:** 2016-11-18

**Authors:** Shimpei Miyamoto, Masahide Fujiki, Nokitaka Setsu, Akira Kawai

**Affiliations:** 1 Division of Plastic and Reconstructive Surgery, National Cancer Center Hospital, 5-1-1, Tsukiji, Chuo-ku, Tokyo 1040045 Japan; 2 Division of Orthopedic Surgery, National Cancer Center Hospital, 5-1-1, Tsukiji, Chuo-ku, Tokyo 1040045 Japan

**Keywords:** Popliteal artery, Revascularization, Flow-through, Lateral circumflex femoral, Bypass flap

## Abstract

**Background:**

Several methods have been reported for intercalary reconstruction of femoral defects. Of these, free vascularized fibula grafts (FVFG) are preferred because of their durability, bone-healing potential, and tolerance to infection. If the bone tumor invades the femoral vessels, simultaneous vascular reconstruction also becomes necessary and significant technical hurdles make limb salvage difficult.

**Case presentation:**

We present a 10-year-old girl who underwent limb-sparing surgery for a distal femur osteosarcoma. The femoral defect was 15 cm long, and the femoral vessel defect was 10 cm long. The femur was reconstructed with bilateral FVFG, and the femoral vessels were reconstructed with saphenous vein grafts. The grafts survived without vascular compromise, and the affected limb was preserved successfully.

**Conclusions:**

Combined use of bilateral FVFG and autologous vein grafts makes limb-sparing surgery for a large osteosarcoma of the femur possible.

## Background

Limb-sparing surgery of a massive femoral defect is a reconstructive challenge. Several methods for bridging a massive femoral defect have been reported, including massive allografts [1], free vascularized fibula grafts (FVFG) [2], massive allografts with FVFG (Capanna method) [3], recycled autografts [4], recycled autografts with FVFG [5], and segmental prostheses [6].

Of these, FVFG is the most suitable procedure because of its durability, bone-healing potential, and tolerance to infection [7]. However, the mechanical strength of single FVFG is not sufficient for femoral reconstruction and a stress fracture incidence rate of 7–16% has been reported [8]. To reconstruct a long femoral defect, bilateral FVFG therefore become necessary. If the bone tumor invades the femoral vessels, simultaneous vascular reconstruction is also required. In these cases, there are significant technical hurdles to salvaging the limb.

To the best of our knowledge, there has been no report of successful reconstruction of a complex femur defect including the femoral artery. We describe a case of a 10-year-old girl who underwent simultaneous reconstruction of the distal femur and femoral vessels using bilateral FVFG and autologous vein graft after osteosarcoma resection.

## Case presentation

**Fig. 1:**
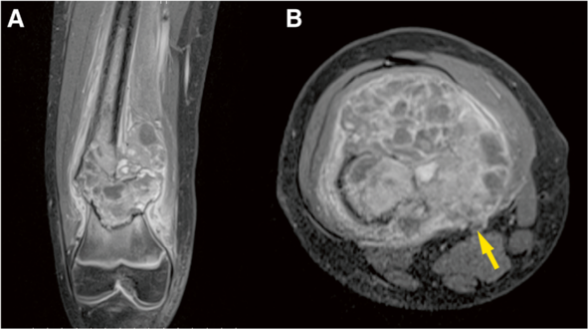
Preoperative contrast-enhanced magnetic resonance image. **a** Coronal view. **b** Axial view. Superficial femoral vessels (*arrow*)

A 10-year-old girl presented with osteosarcoma of her right distal femur, identified after a pathological fracture. Contrast-enhanced magnetic resonance imaging showed a large tumor arising from the distal femur and infiltrating the wall of the superficial femoral artery (Fig. 1). She was otherwise healthy. She underwent neoadjuvant chemotherapy with adriamycin, cisplatin, and methotrexate. Then, intercalary wide resection of the femur, including the femoral artery and vein, was performed. The bony defect was 15 cm long and the vascular defect was 10 cm long. The sciatic nerve and the saphenous nerve were preserved.

**Fig. 2:**
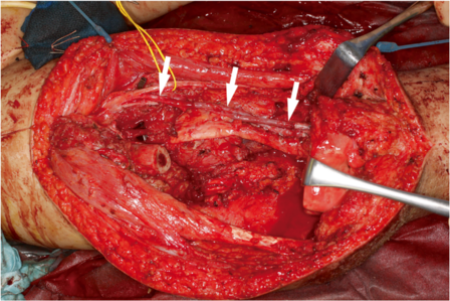
Intraoperative appearance after reconstruction of the superficial femoral artery and vein (*arrows*). Left side is craniad

**Fig. 3:**
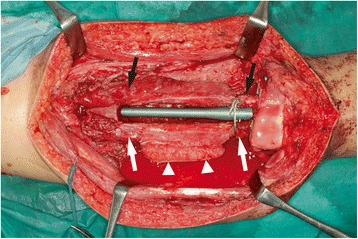
Intraoperative appearance after transfer of the bilateral FVFG. Left side is craniad. left fibula (*black arrows*), right fibula (*white arrows*), and monitoring flap of the right fibula (*arrow heads*)

The ipsilateral great saphenous vein (GSV) was harvested, and the superficial femoral artery and vein were reconstructed with interpositional vein grafts (Fig. 2). The bony defect was bridged with an intramedullary nail, and bilateral fibular grafts were transferred. Both fibular grafts were harvested with a monitoring flap; however, circulation of the left monitoring flap was found to be poor and the flap was sacrificed. The left fibula was hooked up to the superficial femoral artery and vein with an end-to-side anastomosis. The right fibula was hooked up to the descending branch of the lateral circumflex femoral artery and its comitant vein (Fig. 3). The monitoring flap of the right fibula was externalized for postoperative monitoring. We did not perform tibiofibular metaphyseal synostosis for either leg.

**Fig. 4:**
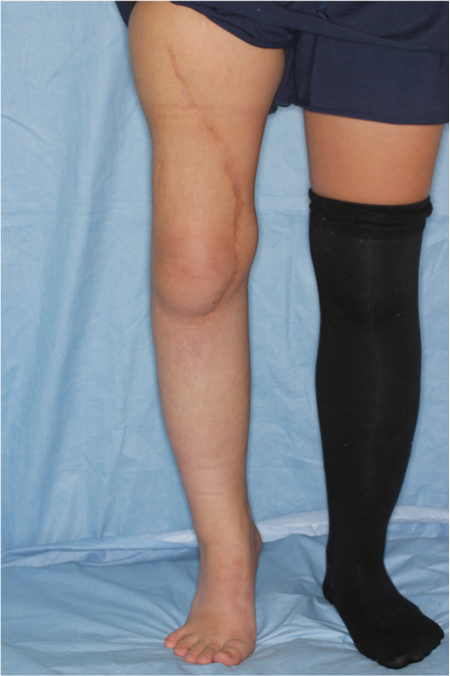
Appearance after 1 year

**Fig. 5:**
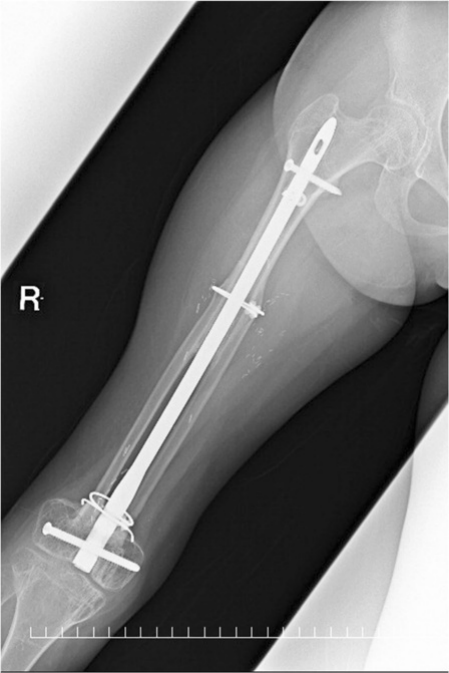
Postoperative X-ray obtained after 20 months shows complete bone union

The wounds healed uneventfully. The circulation of the left fibula was confirmed using color doppler ultrasonography. The rehabilitation started from the fifth postoperative day. The patient underwent adjuvant chemotherapy with the same drugs from the 15th postoperative day for 3 months. At 7 months after the operation, the patient was allowed to walk with full weight bearing. Contrast-enhanced computed tomography obtained 14 months after the operation showed patency of the femoral artery and vein. At 20 months after the operation, the patient was able to walk without any assistance, despite a slight leg length discrepancy (Fig. 4). No donor-site morbidity including valgus ankle deformity developed. A plain radiograph obtained 20 months after the operation showed complete bone union (Fig. 5).

### Discussion

We successfully achieved limb salvage for a complex femoral defect after osteosarcoma resection. The defects of the femur and the femoral vessels were reconstructed with bilateral FVFG and autologous vein grafts, respectively.

Several methods have been reported to date for intercalary reconstruction of femoral defects [1–6, 9]. Among them, the most reasonable method for this case was the Capanna method, which combines FVFG with a massive allograft [9]. In this method, the allograft allows for immediate structural strength and FVFG provides excellent bone-healing potential [10]. However, a bone allograft is not readily available in Japan because of socioreligious reasons [4]. Instead, recycling of devitalized autograft is more widely used [4, 5, 11]. Promising results have been reported with its combined use with FVFG for intercalary femur reconstruction [5], but this method was not indicated for this patient because she had a pathological fracture.

The FVFG was the only biological reconstructive method for this patient. The major problem with FVFG for femoral reconstruction is that single-strut FVFG cannot provide sufficient primary stability for femoral reconstruction [12]. The use of folded FVFG is reported to increase initial strength, but the available length is limited to 13 cm [7]. In this patient, the bone defect was 15 cm long, which exceeds the maximum available length of folded FVFG. Therefore, bilateral FVFG became necessary.

The use of bilateral FVFG for intercalary reconstruction of extensive femoral defects has been reported by several authors [7, 8, 12–14]. High rates of bone union with shorter nonweight-bearing duration are reported with this method. Tomita et al. performed bilateral FVFG for 18 femoral pseudarthrosis patients with large bony defects and achieved bone union in 15 of them (83.3%) [14]. Liang et al. performed bilateral FVFG for 16 patients with massive juxta-articular defects of the distal femur. They reported that primary bone union was achieved in 15 patients (93.8%) and eventual union in all patients (100%) [7]. Niethard et al. performed bilateral FVFG for five patients with an oncologic femoral defect. They reported that bone union was achieved in all patients (100%), including two patients with delayed union [12]. Despite these successes, bilateral FVFG are time consuming and technically demanding compared with other alternatives. This technical hurdle becomes even harder after resecting the femoral vessels because bilateral FVFG require two sets of recipient vessels.

Efficacy of vascular reconstruction in the treatment of extremity sarcoma with vascular involvement is well established [15, 16]. Defects of the femoral artery are most commonly reconstructed using an interpositional GSV graft [15]. Synthetic grafts have a similar patency rate to autologous vein grafts [17], but the need for anticoagulation therapy is a major disadvantage in young patients. Autologous vein grafts, therefore, are preferred for young patients. Although the need for simultaneous venous reconstruction is controversial [18], we performed venous reconstruction with a GSV graft in this patient. No postoperative edema of the distal limb occurred, and the reconstructed vein remained patent through the final follow-up at 20 months.

Donor-site morbidity after fibula harvest is not negligible in children. Some authors have reported progressive valgus ankle deformity after fibula harvest in children [19, 20]. To prevent valgus deformity, tibiofibular metaphyseal synostosis has been recommended in children under the age of ten [19]. On the other hand, Kanaya et al. reported that valgus deformity is inevitable even if tibiofibular metaphyseal synostosis is performed [21]. The indication of tibiofibular metaphyseal synostosis in our patient is controversial because she was just 10 years old at the time of surgery. We did not perform tibiofibular metaphyseal synostosis in this patient, and no ankle deformity developed.

To the best of our knowledge, there has been no previous report of the simultaneous reconstruction of the femoral artery and femur. This paucity can be explained by the suggestion that patients with femoral osteosarcoma with vascular involvement are commonly stratified to above-knee amputation or knee rotationplasty. Several studies demonstrated that, if the tumor infiltrates or surrounds the femoral artery, there is no difference in local tumor control and overall survival between amputation and limb salvage with vascular reconstruction [22–24]. Patients with knee rotationplasty are expected to have almost the same function as those who undergo below-knee amputation [25, 26]. Despite the high functionality, the resultant disfigurement is difficult for girls to accept.

## Conclusions

The combined use of bilateral FVFG and lower limb revascularization with a GSV graft is a somewhat heroic measure, but it can be a permanent and durable limb salvage solution for complex femoral defects. The functional and cosmetic advantages of this method are of great significance in the young population.

## Abbreviations

FVFG: Free vascularized fibula grafts
GSV: Great saphenous vein
